# Bacterial Involvement in Progression and Metastasis of Adenocarcinoma of the Stomach

**DOI:** 10.3390/cancers14194886

**Published:** 2022-10-06

**Authors:** Amanda D. Morgan, Kevin D. Seely, Lauren D. Hagenstein, Garrett M. Florey, James M. Small

**Affiliations:** 1 College of Osteopathic Medicine, Rocky Vista University, Ivins, UT 84738, USA; 2 College of Osteopathic Medicine, Rocky Vista University, Parker, CO 80134, USA; 3 Department of Biomedical Sciences, Rocky Vista University, Parker, CO 80134, USA

**Keywords:** gastrointestinal neoplasm, gastric cancer, gastric neoplasia, metastasis, carcinogenesis, malignancy, bacterial infection, infectious disease, epithelial–mesenchymal transition

## Abstract

**Simple Summary:**

Infectious bacteria influence primary gastric carcinogenesis, organotropism, and metastatic progression by altering the microenvironment at the primary and secondary tumors. Key species include *Helicobacter pylori* (*H. pylori)* and *Mycoplasma hyorhinis (M. hyorhinis).* Inflammation caused by *H. pylori* virulence factors, such as CagA, VacA, and oipA, disrupt epithelial integrity, which allows the primary tumor to progress through the metastatic process. Evidence supports the activation of aquaporin-5 by CagA-positive *H. pylori* infection, promoting epithelial–mesenchymal transition via the extracellular signal-regulated kinase/mitogen-activated protein kinase (MEK/ERK) pathway, thus laying the foundation for metastatic disease. *M. hyorhinis* has also been implicated in gastric neoplasia via β-catenin stabilization and subsequent activation of the WNT-signaling pathway, promoting gastric cancer cell motility and inciting cancer progression.

**Abstract:**

Gastric cancer metastasis is a process in which the tumor microenvironment may carry significant influence. *Helicobacter pylori* (*H. pylori*) infection is well-established as a contributor to gastric carcinoma. However, the role that these bacteria and others may play in gastric carcinoma metastasis is a current focus of study. A review of the literature was conducted to elucidate the process by which gastric adenocarcinoma metastasizes, including its ability to utilize both the lymphatic system and the venous system to disseminate. Studies that investigate the tumor microenvironment at both the primary and secondary sites were assessed in detail. *H. pylori* and *Mycoplasma hyorhinis (M. hyorhinis)* were found to be important drivers of the pathogenesis of gastric adenocarcinoma by modifying various steps in cell metastasis, including epithelial–mesenchymal transition, cell migration, and cell invasion. *H. pylori* is also a known driver of MALT lymphoma, which is often reversible simply with the eradication of infection. *M. hyorhinis* has been implicated in gastric neoplasia via β-catenin stabilization and subsequent activation of the WNT-signaling pathway, promoting gastric cancer cell motility and inciting cancer progression. *Fusobacterium nucleatum* (*F. nucleatum*) and its association with worse prognosis in diffuse-type gastric adenocarcinoma are also reviewed. Recognition of the roles that bacteria play within the metastatic cascade is vital in gastrointestinal adenocarcinoma treatment and potential reoccurrence. Further investigation is needed to establish potential treatment for metastatic gastric carcinoma by targeting the tumor microenvironment.

## 1. Introduction

This literature review is the second of a two-part in-depth literature review on bacterial involvement in gastrointestinal neoplasia metastasis. The first publication focused on bacterial involvement in colorectal carcinomas [1]. The current review examines the literature on gastric carcinoma and the bacterial drivers of its oncogenesis and metastasis. 

Gastric adenocarcinoma was the fifth most common cancer and the fourth leading cause of cancer-related deaths worldwide in 2020 [2]. There were an estimated 1.1 million new cases and 770,000 gastric cancer-related deaths worldwide in 2020 [3]. Morgan et al. project that if the current rates remain stable, 1.8 million cases and 1.3 million deaths are expected to occur in 2040, which is 66% and 71% higher than that estimated in 2020, respectively [3]. It is known that greater than 90% of cancer-related deaths are caused by metastatic disease, which remains a significant clinical challenge in oncology [4]. The vast majority (90%) of non-cardia gastric neoplasms are attributable to *Helicobacter pylori (H. pylori)* infection [5].

While much has been written about the relationship between *H. pylori* infection and carcinogenesis, little has been written about *H. pylori’s* involvement in metastasis of gastric cancer. There is minimal research on the bacteria that may contribute to metastatic disease, and it is still unknown whether a tumor depends on the same microbial microenvironment before, during, or after hematogenous or lymphatic spread. While numerous associations between viruses, bacteria, parasites, and carcinogenesis have been made, it is a relatively new concept that specific microbial drivers might influence both primary tumors and their metastases. We selected gastric cancer as the entity of focus for this review because the interrelationship between *H. pylori* and gastric neoplasia is well established. 

Lauren’s classification system for gastric adenocarcinoma is reviewed in Section 2 to orient the reader. Section 3 discusses the most common site of metastasis of gastric neoplasia, including a discussion on Krukenberg tumors and peritoneal seeding. We then review the literature to characterize gastric neoplasia tumor microenvironments (Section 4). In Section 5, we analyze the literature to detail the role of bacteria in gastric neoplasia metastasis, and the microenvironment of both primary and secondary sites of tumor proliferation. The literature on tumor microenvironment considerations in treatment and gastrectomy as the treatment for confirmed gastric CA is reviewed in Section 6. The central aim of this review is to add clarity and contribute to the understanding of the role of bacteria in the progression of neoplasia and metastatic disease and to uncover novel potential points of treatment augmentation.

## 2. Colorectal Neoplasia and Metastasis

The development of the Lauren classification system for gastric carcinomas in 1965 changed the approach to gastric cancer. Despite the varying causes of gastric adenocarcinoma, medical professionals were able to differentiate the tumors based on histology (general structure, cell structure, mucosal secretion, and mode of growth) into one of two types, intestinal type or diffuse type [6].

The intestinal type has distinct, large glandular lumina, which contain papillary folds. The cells of the intestinal type were characterized by their larger size, variability, and defined borders. Of special note during the study, there was little mucus secretion, and the growth of the tumor was clear and defined. It was understood that the intestinal-type carcinoma did not infiltrate past the defined border of the tumor.

Conversely, the diffuse type is described by clusters of cells that are scattered with small glandular lumina as its general structure. Often, there are prominent mucus droplets, giving rise to the descriptive name of “signet ring carcinoma.” The cell’s structure is remarkable for its fragile cytoplasm and uniformity amongst the cells. Mucus secretion was extensive in the diffuse-type gastric carcinomas and the mode of growth was wider than in the intestinal type [6]. The diffuse type is sometimes referred to as Linitis Plastica. These features provided a needed guideline that propelled the Lauren classification into medical practice and treatment decisions. In general, the intestinal-type carcinoma is more common in males and older individuals, whereas the diffuse type has a poorer prognosis overall [6]. A more recent study elaborated on the Lauren classification, with an emphasis on including a third type into the classification, with the three types being mesenchymal, proliferative, and metabolic [7]. The third type has not been widely accepted in current treatment protocols. The College of American Pathologists’ cancer protocol recommends using the world health organization classification of gastric adenocarcinoma, which builds upon Lauren’s criteria [8].

## 3. Metastasis Sites of Gastric Neoplasia

**Figure 1:**
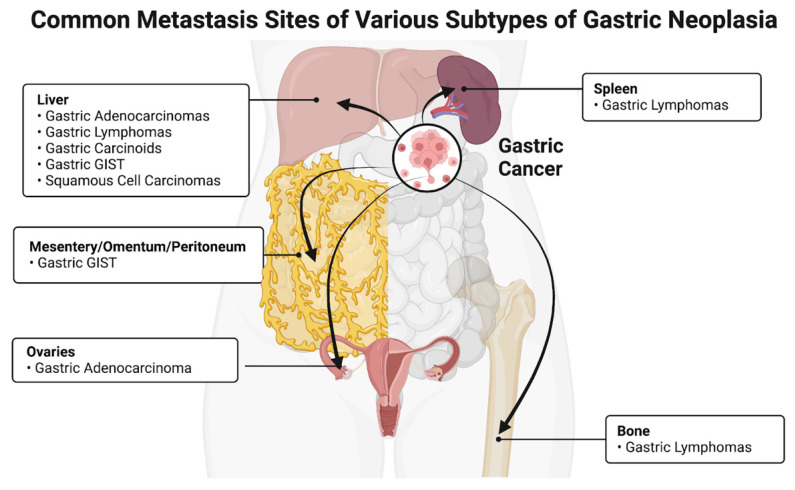
The most common metastasis sites for gastric cancer include the liver, mesentery/omentum, spleen, and bone. Figure created using biorender.com.

Gastric neoplasia includes adenomas, lymphomas, gastrointestinal stromal tumors (GIST), mucosa-associated lymphoid tissue (MALT) lymphoma, and carcinoid tumors. An interesting note about these cancers is their relative similarities in the secondary metastasis sites. After regional lymph nodes, gastric and colorectal cancers most commonly metastasize to the liver, non-regional lymph nodes, the lung, and peritoneum [9] (Figure 1). Unlike most gastrointestinal carcinomas, a key feature of gastric neoplasia is metastasis to the pleura and peritoneum [9]. Gastric adenomas, gastric lymphomas, gastric adenocarcinoma, GIST, and gastric carcinoids are significant in the discussion of gastrointestinal cancer. Riihimaki et al. found that gastric adenocarcinomas most commonly metastasize to the liver (53%), pleura/mediastinum (28%), bone (17%), and nervous system (11%) [10]. Gastric lymphomas were reported to spread to the liver, bone, and spleen [11]. In another study, GIST was found to metastasize most commonly to the liver (28%), and mesentery/omentum (30%) [12]. Lastly, gastric carcinoids were found to mostly metastasize to the liver [13].

An important distinction to establish is the difference between cardia gastric neoplasia and non-cardia gastric neoplasia, which are named based on their predominant anatomic location within the stomach. Cardia gastric CA is related to Barrett’s metaplasia and is thought to be a result of chronic inflammation and acid reflux. It commonly metastasizes to the lungs, bone, and nervous system. Non-cardia gastric cancer is the predominant type, is the prototype for Lauren’s criteria, and is the main topic of discussion in this review. Non-cardia gastric cancer metastasizes more commonly to the peritoneum [10]. One study showed a strong positive association between *H. pylori* infection and non-cardia gastric cancer, and a statistically significant negative association with cardia cancer [14]. These patterns of metastasis, which differ depending on the cancer type and location, hold significant value in the understanding of the tumor microenvironment and metastatic mechanism. 

Furthermore, different metastasis patterns depend on the origin site and the specific type of carcinoma (Table 1). Various studies have been conducted to delve deeper into the role of the venous system in the metastatic cascade of gastric tumors. It is hypothesized that this spread occurs through circulating tumor cells (CTCs), which travel through hematogenous spread to distant sites of the body [4]. Li et al. illustrated the potential of applying CTCs to understand the severity and spread of advanced gastric cancer [15]. This study demonstrated that higher numbers of CTCs were found in patients with primary gastric cancer, compared to gastroesophageal cancer. Patients who exhibited lower CTC counts or converted to lower CTC counts throughout treatment had more favorable outcomes in terms of overall survival and progression-free survival. Patients with high initial CTC counts who had worse overall survival, despite not demonstrating progressive gastric cancer in terms of tumor diameter, indicate that CTCs are a potential biomarker that can be used to understand the severity of gastric cancer.

DNA fragments that arise from the primary tumor circulate in the blood and can be detected, isolated, and sequenced [16]. These fragments can provide insight into potential genetic mutations that the patient harbors, such as a TP53 mutation [17] and HER2 amplification [18]. Patients with increased circulating tumor DNA (ctDNA) levels in advanced-stage gastric cancer correlated with a lower five-year survival rate and worse prognosis [19]. 

### 3.1. Krukenberg Tumors and Diffuse-Type Gastric Adenocarcinoma

The Krukenberg tumor is a metastatic mucinous signet ring adenocarcinoma that often spreads to bilateral ovaries [20]. Studies have demonstrated that this tumor most commonly metastasizes from a gastric adenocarcinoma primary tumor (70%) [21]. However, some research has indicated that colorectal carcinoma may also be a common site of the primary tumor [22]. The proposed mechanism of metastasis is a large focus of current research. The three dominant theories on how the gastric tumor spread to the ovaries are either hematogenous, lymphatic, or peritoneal spread [23]. Lymphatic spread is the leading theory for how this process occurs, due to the extensive lymphatic network within the gastric mucosa and submucosa [21]. There also seems to be some association between the extent of lymph node involvement and ovarian metastasis, although the two are not directly correlated in mechanism [24].

Wang et al. found that during the metastasis process, specific biomarkers remain constant, such as HER2/neu, c-met, p53, and ki67, between the primary gastric tumor and the Krukenberg tumor [25]. This is a potential avenue for researchers to explore and understand how the metastasis process occurs. 

No association has been found between bacteria within the primary tumor location and the Krukenberg tumor. Future research must be conducted to analyze if there is any connection that would associate the primary gastric tumor microenvironment to the secondary tumor within the ovarian microenvironment.

### 3.2. Epithelial-Mesenchymal Transition in Gastric Adenocarcinoma Metastasis

Adenocarcinoma metastasis involves the breakdown transmembrane glycoproteins that mediate intercellular adhesion and signaling, such as E-cadherins and beta-catenin, which are responsible for maintaining the connections between epithelial tumor cells in the primary tumor [26]. Diminished E-cadherin function underlines the pathogenesis of many epithelial tumors, including adenocarcinoma of the stomach [27]. Furthermore, loss of E-cadherin underlines advanced tumor stage and a poor prognosis [28]. The Wnt signaling pathway and cell–cell adhesion are both impacted by the disruption of the E-cadherin/beta-catenin complex. More specifically, cellular adhesions break down and the cells dissociated when E-cadherin is downregulated, inhibited, or eliminated [29,30]. 

Epithelial-mesenchymal transition (EMT) is the process by which epithelial cells are transformed into mesenchymal cells. EMT is thought to be the mechanism by which E-cadherin expression is silenced in some cancers [31,32,33]. It is a complex biological process that has been identified as a key component of carcinogenesis because EMT-derived tumor cells exhibit stem cell traits, proliferate quickly, and are extremely resistant to therapy [34]. EMT and metastasis are influenced by numerous kinase-mediated signaling pathways, some of which are triggered by bacterial infection. For instance, in the context of IL-10 deficiency, *Enterococcus faecalis* has been connected to the TGF-1/Smad signaling pathway in murine studies [35].

## 4. Tumor Microenvironments

The tumor microenvironment of GI cancers has been increasingly studied in recent literature. This microenvironment contains elements (fibroblasts, endothelial cells, pericytes, macrophages, stem cells, and invasive cells) that surround or are within the tumor to aid in survival mechanisms [36,37]. For example, the microenvironment has the ability to promote angiogenesis, invasion, and metastasis within the human body [38,39,40]. With an understanding of a tumor’s microenvironment, there is a strong possibility of predicting the mechanisms of cancer and potentially targeting the tumor microenvironment in future cancer treatments. Given the growing importance and understanding of the tumor microenvironment in cancer biology, cancer research and treatment have shifted from a cancer-centric model to a microenvironment-centric approach. However, the clinical effectiveness of therapeutic approaches that target the microenvironment of tumors, particularly the cells or pathways of the microenvironment itself, is not yet satisfactory [41]. 

Gastric neoplasia is often driven by chronic inflammation, which, in theory, changes the microenvironment of the tissue to support more inflammatory cells. This change in the tumor microenvironment promotes carcinogenesis. Tumor-associated macrophages have immunosuppressive effects that also contribute to the evolving tumor microenvironment of gastric cancers. In gastric cancer, Cadherin 11 was shown to be associated with the transformation of macrophages in the tumor microenvironment, which tilts the microenvironment towards an immune-based environment [42].

Autoimmune gastritis (AIG) is an autoimmune condition that specifically affects the fundus and body of the stomach and is still a field of evolving research, including exploring the degree that gut microbiota has an impact on its development and prognosis [43]. As the acid-secreting parietal cells are destroyed, the well-established sequelae are iron-deficiency anemia and gastric cancer [44]. This paper discusses the importance of gastric acidity in inhibiting detrimental microbiota growth. It is worth noting that fully identifying the microbial content of the stomach involves several hurdles to overcome, such as contamination from the oropharynx, inability to culture the vast majority of organisms, and normal variations in stomach acidity, amongst many other factors [45,46]. Although there have been studies published that look at the possible changes in microbial composition in AIG, these have been generally inconclusive, with any changes being attributed to the hypochlorhydria state allowing oral bacteria to migrate distally [47,48,49]. One study showed that patients with AIG had increased microbial burden and diversity, with particular increases in *Streptococcus* populations, which is suggestive of oral migration [50].

## 5. Bacterial Involvement in Gastric Carcinogenesis

Certain microbial species are associated with specific neoplastic patterns [51]. For example, *H. pylori* is a known cause of MALT lymphoma and gastric adenocarcinoma [52,53], while *Streptococcus bovis* is associated with colorectal cancer [54]. This review focuses on neoplasms of the stomach, which are among the most common significant causes of cancer morbidity and mortality in the world. It was previously shown that the incidence and mortality rates of gastric cancer have declined since the recognition of and ability to eradicate *H. pylori* infection. This trend evidently has reversed in recent years, with some research suggesting that the rates of stomach cancer may be increasing amongst younger age groups in the United States [55]. Three important bacterial species have been identified in the pathogenesis and metastasis of gastric adenocarcinoma, including *H. pylori, M. hyorhinis*, and *F. nucleatum* (Table 2). Although others are under investigation, the aforementioned three are certainly the most notable and the most dangerous and therefore will constitute the species of concern in this review.

### 5.1. Helicobacter pylori

**Figure 2:**
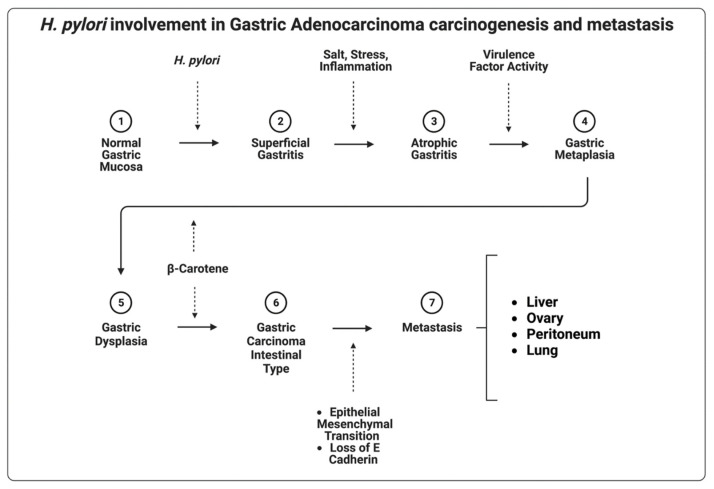
Gastric adenocarcinoma begins as superficial gastritis and can progress into atrophic gastritis, metaplasia, dysplasia, and gastric adenocarcinoma, followed by metastatic disease as the feared outcome. Superficial gastritis due to *H. pylori* is more likely to lead to intestinal type gastric adenocarcinoma.

*H. pylori* are spiral-shaped, Gram-negative, urease-positive bacteria, with polar flagella that inhabit the inner lining of the gastric epithelium of humans [62]. The species is the first and only bacterium classified as a group A carcinogen by the International Agency for Research on Cancer [63]. If left untreated, *H. pylori* causes gastritis [64], peptic ulcer disease [65], MALT lymphoma [52], and gastric adenocarcinoma [53]. The proposed mechanism for the development of gastric adenocarcinoma in the setting of *H. pylori* infection involves increased gastric epithelial cell proliferation in the background of chronic inflammation (Figure 2) [66]. *H. pylori* are non-invasive, but infection with *H. pylori* creates an inflammatory environment that contains genotoxic agents, such as reactive oxygen species [67]. Histologically, *H. pylori* gastritis is often chronic active gastritis with a mix of mononuclear and neutrophilic leukocytes [68]. Additionally, the *H. pylori* genome contains genes directly implicated in oncogenesis. Cytotoxin-associated A (CagA) gene [69], vacuolating cytotoxin A (VacA) gene [70], and outer-inflammatory protein A (oipA) [71] gene are commonly cited as causes of serious disease [72]. CagA-expressing isolates of *H. pylori* colonize the gastric mucosa and induce proinflammatory cytokine secretion, atrophy, and subsequent intestinal metaplasia.

Over half of the world’s population carries *H. pylori* [73]; however, not all develop the serious associated sequelae. Why *H. pylori* infection does not cause ulcers in every infected person is unknown. The relationship between *H. pylori* virulence factors and host immunity is one element that determines the fraction of infected individuals who will develop serious illnesses [74]. Genetic polymorphisms that favor the expression of the proinflammatory cytokine tumor necrosis factor (TNF) [75] and interleukin-1 *beta* (IL-1β) [76], or decrease expression of interleukin-10 (IL-10) [77], are associated with increased development of pangastritis and sequelae. Virulence factors specific to the isolate are key determinants of outcome. For example, the CagA gene is present on 50% of isolates overall, but in 90% of *H. pylori* isolated in populations with an increased prevalence of gastric adenocarcinoma [78]. 

Other factors that determine the severity of disease progression in some individuals and not in others are ongoing areas of research. For example, *H. pylori* are detected in the gastroduodenal mucosa in the majority of patients with duodenal ulcers, but only a minority (10 to 15%) of *H. pylori*-infected patients develop peptic ulcer disease [79,80]. It is usually curative of peptic ulcer disease if successfully eradicated, and re-infection after eradication is rare [81]. It is likely that successful detection and eradication contribute to the low rates of disease progression among infected individuals. 

Human gastric carcinogenesis is a multi-step and multifactorial process. This process begins as superficial gastritis and progresses into atrophic gastritis, metaplasia, dysplasia, and then gastric adenocarcinoma, in a manner first described by Corea et al. in 1988 [82,83]. Constituent Wnt/β-catenin pathway activation via upregulated expression of Wnt10a and Wnt10b by *H. pylori* virulence factors has been identified as a common mutation that drives this progression [84,85]. Evidence also supports the activation of aquaporin-5 by CagA-positive *H. pylori* infection, which promotes epithelial–mesenchymal transition via the extracellular signal-regulated kinase/mitogen-activated protein kinase (MEK/ERK) pathway, thus laying the foundation for metastatic disease [86]. 

*H. pylori* are also associated with extra nodal marginal zone B-cell lymphoma, also known as mucosa-associated lymphoid tissue (MALT) lymphoma [87]. MALT is not present in normal gastric mucosa, but can be induced in the setting of chronic gastritis. Induced gastric MALT is most commonly a result of *H. pylori* infection [56]. Three translocations linked to gastric MALT lymphoma lead to increased expression of intact MALT1 and BCL-10 proteins. NF-B, a transcription factor that promotes B-cell growth and survival, is constitutively activated as a result. BCL-10 and MALT-1 are necessary for the antigen-dependent activation of NF-B in healthy B and T cells. They collaborate in a signal transduction pathway downstream of lymphocyte antigen receptors, and their constitutive activation is pro-oncogenic [88]. *H. pylori* can evidently stimulate NF-κB activation through the MALT1/BCL-10 pathway in MALT lymphomas that lack inciting translocations [89]. This hypothesis is further supported by the fact that tumors that lack translocations are resolved with *H. pylori* eradication, but tumors with translocations that involve MALT1 or BCL-10 persist after eradication [90]. MALT lymphomas that accumulate inactivating mutations of tumor suppressor genes that encode for p53 or p16, for example, can become more aggressive tumors that are not amenable to *H. pylori* eradication [91]. Current research on *H. pylori* focuses on its epidemiology [2,73], improving testing [92], treatment [57], and a better understanding of its unique virulence factors [58], and its ability to propel progression [86]. Primary prevention through eradication of *H. pylori* and lifestyle modifications, including reducing salt intake, smoking, obesity, and alcohol, remains key in gastric CA control [3]. Additionally, eating foods that have been salted or smoked is associated with developing diffuse-type gastric adenocarcinoma. The biosynthesis of glycolipids in the cell wall of *H. pylori* is thought to be inhibited by glycans found in glandular mucus, and a high-salt diet (regular consumption of sodium beyond recommended daily value) is thought to increase superficial mucus cell mucus, while decreasing glandular mucus cell mucus [93].

### 5.2. Mycoplasma hyorhinis

*Mycoplasma hyorhinis (M. hyorhinis)* are Gram variable cell wall-deficient bacteria. This small Gram-negative pleomorphic coccobacilli is known to infect the respiratory tract of pigs. Numerous swine diseases have been linked to *M. hyorhinis*, with severe pathogenicity, high mortality rates, and ensuing financial losses. Antibiotic interventions to lessen these effects encourage the growth of drug-resistant *M. hyorhinis* strains which have the potential to infect human stomachs through pork consumption [94]. A previous report identified *M. hyorhinis* in 56% of gastric cancer [95].

Gastric and prostate neoplasms have been linked to *M. hyorhinis* dysbiosis via the NLRP3 inflammasome [96]. NLRP3 is a protein complex that regulates the maturation of pro-inflammatory cytokines, such as interleukin-1 (IL-1) and interleukin-18 (IL-18), and is also involved in tumorigenesis and metastasis. Mounting evidence suggests that *M. hyorhinis* infection results in pathology in human studies. Serology studies have confirmed *M. hyorhinis* in gastric carcinoma, colon carcinoma, and prostate and lung carcinoma biopsies [59,95].

A 2019 study by Liu et al. revealed *M. hyorhinis* involvement in gastric neoplasia via β-catenin stabilization and subsequent activation of the WNT-signaling pathway, promoting gastric cancer cell motility and inciting cancer progression [59]. They found that when glycogen synthase kinase 3 beta (GSK3β) and Wnt-receptor lipoprotein-receptor-related protein 6 (LRP6) did not interact, there was no increase in activating β-catenin stabilization, suggesting that this interaction has a carcinogenic effect. They also showed an interaction between LRP6 and p37, a mycoplasma membrane protein known to have carcinogenic effects [97]. In vitro studies have found p37 to promote cell motility, migration, and invasion through the activation of metalloproteinase-2 and epidermal growth factor receptor [60], veritably connecting *M. hyorhinis* infection and metastatic disease.

### 5.3. Fusobacterium nucleatum

*Fusobacterium nucleatum (F. nucleatum)* is an opportunistic, Gram-negative obligate anaerobic bacterium which is commonly located in the oral cavity of humans [98,99]. *F. nucleatum* is frequently detected in primary colorectal cancer (CRC) and its metastases, and has been linked to a worse prognosis in gastrointestinal cancers in general. Recently, studies have shown increased loads of *F. nucleatum* in gastric cancer tumor samples [94], although it is unclear if it is a causative agent. In one study, *F. nucleatum* positivity showed no association with chronic gastritis or preneoplastic conditions, such as intestinal metaplasia [61].

*F. nucleatum* is associated with worse prognosis in Lauren’s diffuse-type gastric cancer patients, but not in the intestinal type, in contrast to *H. pylori* [61]. *F. nucleatum* increases cell proliferation and tumor-promoting inflammation, while avoiding immune destruction, ultimately promoting a pro-inflammatory state and tumorigenic environment [100,101]. *F. nucleatum* is thought to stimulate cell proliferation via two primary signaling mechanisms, including (1) FadA binding to E-Cadherin to activate the WNT/B catenin pathway [102] and (2) interacting with the Toll-like receptor 4 (TLR-4) to activate P21-activated kinase 1 (PAK 1), a protein that phosphorylates the B-catenin pathway [103,104]. However, these patterns have only been established in CRC studies, and further studies are needed to explore the related mechanistic insights and potential therapeutic benefits of targeted antibiotic treatment in gastric cancer patients.

## 6. Considering the Microbiome in Gastrointestinal Cancer Treatment

Investigations into the complex interactions of microorganisms and tumor behavior are uncovering a variety of potential mechanisms by which infection/colonization contributes to more aggressive tumors. Factors such as increased acute and chronic inflammation, induction of various enzymes, stimulation of cytokines, changes in the microbiome, and even potential alterations in local oxygen tension may modulate the growth, invasiveness, and spread of neoplastic cells.

Tumor cells may degrade interstitial connective tissue and the basement membrane by secreting proteolytic enzymes or by inducing stromal cell proteolysis [105,106]. Ulceration or chronic inflammation in the setting of ulcerative colitis or bacterial infection could affect the process of basement membrane degradation, catalyzing this cascade step [65,107]. Matrix metalloproteinases (MMPs), amongst others, have been implicated in tumor cell invasion [108]. Both *H. pylori* and *M. hyorhinis* have been shown to induce metalloproteinase activity [60,109]. MMPs remodel the basement membrane and interstitial connective tissue to promote progressive invasion and metastasis [110]. They also promote metastasis by inducing factors related to malignant behavior. MMP-2 and MMP-9 have been linked to vascular endothelial growth factor (VEGF), a necessary compound for tumor vascularization [111].

It is important to note that the regions within the stomach have varying microflora compositions [112]. Eradication of *H. pylori* has been repeatedly shown to decrease the risk of neoplasia, especially when treated before pathologic site changes have occurred and even in those with prior gastric cancer history [113]. Prognosis is most accurately determined after thoroughly investigating the cells and microenvironment. Gastric cancers are often the culmination of lifestyle factors, such as smoking, diet, and Epstein–Barr virus infection [114], but that is not to say mutations are not important in tumorigenesis [115]. For example, a study found that gastric cancers often have a lower relative expression of NFKB2, which is part of a family of transcription factors [116].

However, bacterial consideration does not end there. A study showed detrimental gut bacteria such as *Proteobacteria* flourished after *H. pylori* eradication, which may warrant probiotic consideration in the treatment plan [117]. Bik et al. showed an overabundance of *Propionibacterium acnes*, *Streptococcus anginosus*, and *Prevotella melaninogenica* in the gastric tumoral microenvironment, which are associated with gastric cancer tumorigenesis through their specific mechanisms that increase local inflammation [118]. *P. acnes* releases short-chain fatty acids, which can cause lymphocytic gastritis. *S. anginosus* induces cytokine release via sulfur metabolism. *P. melaninogenica* contributes to excess gastric acidity in non-atrophic sites [119,120,121]. Identifying microbes that are similar to these and their pathways of pathogenicity is crucial for developing targeted treatment plans.

### Microbiome after Gastrectomy

For early-stage tumors, gastrectomy is the most successful definitive treatment. A landmark study in 1982 recommended excision margins of at least six centimeters to properly excise serosa infiltrations and constituted the standard of treatment for nearly 30 years. However, increasing the amounts of gastric tissue removed leads to poorer patient quality of life and nutrition [122,123]. Many studies since then have tried to further define the most appropriate proximal resection margin (PRM) with conflicting results; a 2017 study recommended at least 2.1 cm of PRM [124]. In an attempt to provide a definitive answer, a 2020 retrospective analysis concluded that previously recommended PRMs may not be absolute, with distances as short as < 1 cm providing comparable rates of survival [125]. Furthermore, patients with larger-end PRMs may have partial gastrectomy that effectively provides the physiology of complete gastrectomy.

Many studies have analyzed the alteration in the microbiome post-gastrectomy. The Roux-en-Y gastrojejunal anastomosis (RYGJ) and Billroth II anastomosis (BII) are two types of partial gastrectomy that may be performed for neoplasm resection and are shown to alter gut microbiota. One study showed *Ralstonia* and *Helicobacter* predominating before and *Streptococcus* and *Prevotella* predominating after tumor resection [126]. Another study found an increased abundance of *Anaerosinus*, *Butyrivibrio*, *Campylobacter*, *Clostridium*, *Coprococcus*, *Desulfovibrio*, *Oscillospira*, *Oxalobacter*, *Slackia*, *Sporobacter*, *Veillonella*, and *Victivallis* after a BII or RYGJ, compared to controls without these surgeries [127]. Although the RYGB is commonly performed for weight loss, the evidence suggests that microbial alterations are more likely driven by direct changes to intestinal physiology, rather than weight loss, which can likely be also applied to BII [128]. 

Higher concentrations of dissolved oxygen in the hindgut after an RYGJ are thought to lead to an increase in facultative anaerobes, such as *E. coli*, *K. pneumoniae*, *E. faecalis,* and Streptococcus species [128]. Both the RYGJ and BII reduce the stomach luminal surface area, which in turn decreases acid secretions. This creates a less hostile environment for E. coli, promoting its colonization [129]. As a result of decreased acid secretion, the increased stomach pH permits oral flora, such as *Streptococcus* spp. and a few *Veillonella* spp., which are metabolically dependent on *Streptococcus* spp. in oral biofilms, to overcome the previously inhibitory gastric barrier [130]. As with PPIs and sleeve gastric bypass, which typically creates a permanent pH of 6.0, gastric pH levels that surpass 4.0 have significantly diminished antimicrobial effects [131,132]. There are also factors in addition to oxygen concentration and pH that likely affect microbial colonization.

We have gained insight by comparing alterations in the microbiome after partial gastrectomy for cancer and for morbid obesity. The majority of partial gastrectomies are performed to resect tumors. So, the question remains whether tumors found post-gastrectomy can truly be attributed to bacteria-induced inflammatory changes versus recurrence.

Furthermore, partial gastrectomy to treat morbid obesity yields different alterations. In one study, *Yokenella regensburgei* and *Fusobacterium varium* were found after bariatric surgery; these two, in particular, have already been associated with colonic inflammation, which can be theorized to occur in the stomach as well [133]. Evidence supports bariatric surgery being protective against obesity-related cancers, as defined by the International Agency for Research on Cancer (IARC), including esophageal adenocarcinoma, postmenopausal breast cancer, renal, colon, rectum, gastric cardia, liver, gallbladder, pancreas, ovary, corpus uteri, thyroid, multiple myeloma, and meningioma [134,135,136]. However, a study on delineated non-obesity-related carcinomas found that there was only a protective effect of bariatric surgery in females [135]; the sample size was vastly different between males and females, so this could be an area that warrants further research. It is important to note that early-onset gastric cancer is a distinct disease with worrisome trends and oncogenic features and unique clinical and genomic characteristics [137]; therefore, further study is needed to elaborate on bacterial involvement in this entity. The differences in microbiota may have a role in neoplasia protection post-gastrectomy.

## 7. Conclusions

Bacterial involvement in gastric neoplasia and metastasis is significant. Primary tumors are frequently curable with gastrectomy, when detected early. However, metastatic disease, which accounts for the majority of cancer-related deaths, remains a deadly clinical scenario. Growing evidence indicates that bacterial infection influences organotropism and metastatic progression by altering the microenvironment at the primary and secondary tumors, in addition to promoting carcinogenesis in primary gastric CA. Eradication of *H. pylori* can halt gastric MALT lymphoma progression and reverse early metaplasia. Inflammation and disruption of epithelial integrity, brought on by virulence factors such as CagA, VacA, and oipA, enable the primary tumor to go through the critical stages of the metastatic process. The evidence supports the activation of aquaporin-5 by CagA-positive *H. pylori* infection, which promotes epithelial–mesenchymal transition via the extracellular signal-regulated kinase/mitogen-activated protein kinase (MEK/ERK) pathway, thus laying the foundation for metastatic disease. *M. hyorhinis* has been implicated in gastric neoplasia via β-catenin stabilization and subsequent activation of the WNT-signaling pathway, promoting gastric cancer cell motility and inciting cancer progression. *F. nucleatum* is a driver of gastrointestinal cancers in general, and further investigation into its involvement in gastric cancer is needed. *F. nucleatum* is associated with worse prognosis in diffuse-type gastric adenocarcinoma. Recognition of the roles that bacteria play within the metastatic cascade is vital in gastrointestinal adenocarcinoma treatment and potential reoccurrence. At this time, further investigation is needed to establish potential treatment for metastatic gastric carcinoma by targeting the microbial contribution to the tumor microenvironment.

## Figures and Tables

**Table 1:** Gastric neoplasia subtypes listed with their most common metastasis sites.

Cancer Type	Most Common Site	Reference
Adenocarcinoma	Liver, ovaries	[10]
Lymphomas	Spleen, bone, liver	[11]
Gastrointestinal stromal tumors	Mesentery, omentum, ovaries	[12]
Carcinoids	Liver	[10]

**Table 2:** Overview of bacteria associated with gastric neoplasia.

Bacteria	Proposed Pathogenesis	References
*H. pylori*	Increased cellular proliferation and signaling, loss of E-cadherin, β-catenin stabilization, and subsequent activation of the WNT-signaling pathway via virulence factors	[56,57,58]
*M. hyorhinis*	P37 induction and activation of MMP-2, promotes gastric cancer cell motility via β-catenin stabilization and subsequent activation of the WNT-signaling pathway	[59,60]
*F. nucleatum*	Associated with worse prognosis in Lauren’s diffuse-type gastric cancer patients	[61]
